# Comparison of Outcome Between Neoadjuvant Chemotherapy with Limb Salvage Surgery and Amputation with Chemotherapy in Osteosarcoma Patients: A Systematic Review

**DOI:** 10.5704/MOJ.2603.009

**Published:** 2026-03

**Authors:** J Rudyan, IGE Wiratnaya, P Astawa

**Affiliations:** Department of Orthopaedics and Traumatology, Udayana University, Denpasar, Indonesia

**Keywords:** osteosarcoma, chemotherapy, limb salvage surgery, amputation

## Abstract

**Introduction:**

Osteosarcoma is a rare malignant bone tumour arising from primitive mesenchymal tissue, most often affecting males aged 10–30. Historically, treatment involved amputation or disarticulation, with a 5-year overall survival rate of just 20%.

**Materials and methods:**

This systematic review included studies from MEDLINE, Cochrane, EMBASE, and Google Scholar. Additionally, reference lists of relevant sources were manually reviewed. The search, conducted through 2021, used terms such as amputation, osteosarcoma, bone cancer, recurrence, and metastasis.

**Results:**

Eight relevant studies were thoroughly reviewed, most conducted at single institutions and published between 1990 and 2019, covering data from 1983 to 2015 and involving 1,389 patients. Three studies (646 patients) assessed 5-year disease-free survival (DFS) in limb osteosarcoma, reporting a median DFS of 56.9 months (range: 6–78 months). Five studies (969 patients) analysed local recurrence, showing higher relapse rates with limb salvage surgery (LSS) compared to amputation (73.5% vs. 28.1%). Additionally, six studies (1,141 patients) evaluated 5-year overall survival, with better outcomes in the LSS group (351 survivors) compared to the amputation group (248 survivors).

**Conclusion:**

In conclusion, this thorough review makes a valuable contribution to the current understanding of survival and local recurrence outcomes in patients with limb osteosarcoma treated with either LSS or amputation. The findings suggest that LSS is associated with a higher 5-year survival rate compared to amputation.

## INTRODUCTION

Osteosarcoma is an infrequent form of malignant tumour originating from primitive mesenchymal tissue responsible for bone formation. Its prevalence primarily affects young males aged 10 to 30 years old. Among malignant primary bone tumours, osteosarcoma ranks the most prevalent, accounting for approximately 20% of all cases^1,2^. This tumour type primarily emerges in the femur, tibia, and humerus^3,4^. Before the advent of neoadjuvant chemotherapy, prevailing treatments for osteosarcoma encompassed amputation and disarticulation, resulting in a mere 5-year overall survival (OS) rate of about 20%^4,5^.

In current practice, osteosarcoma treatment comprises five drugs: high-dose methotrexate (HDMTX) coupled with leucovorin rescue, doxorubicin, cisplatin, ifosfamide, and etoposide. These medications align with the National Comprehensive Cancer Network's 2020 Guidelines for Osteosarcoma Management^3^. The introduction of effective neoadjuvant chemotherapy during the 1970s marked a shift, making LSS a viable option for osteosarcoma treatment. As chemotherapy regimens advanced and new drugs like cisplatin and ifosfamide complemented high-dose methotrexate, doxorubicin, and bleomycin cyclophosphamide dactinomycin (BCD), the prospects of LSS improved^6^. This approach, often combined with neoadjuvant or adjuvant chemotherapy, offers functional and physiological advantages over traditional amputation, leading to noteworthy enhancements in long-term survival and limb salvage rates and the evolution of diverse surgical techniques^7,8^.

Historical research delineates LSS as fitting for localised osteosarcoma, while amputation suits the aggressive malignant type^9,10^. Nevertheless, some surgeons uphold the belief that swift, comprehensive tumour resection is necessary to stave off fracture-induced progression, thus advocating for amputation in osteosarcoma patients with pathologic fractures^10,11^. Besides, amputation will alter the functional outcome of these patients and may hinder Activities of Daily Living (ADL). Interestingly, studies reveal comparable risk of local recurrence and five-year OS rates between LSS and amputation in osteosarcoma patients with pathological fractures^12^, while others indicate a bleaker prognosis for such cases^13,14^. Contention surrounds the survival and functional recovery advantages LSS versus amputation in osteosarcoma patients^10,11^.

This study delves into whether LSS contributes to improved survival through five-year rates and diminished local cancer recurrence among patients treated with LSS or amputation^14^. Conducted as a meta-analysis, the study seeks to consolidate findings across abundant osteosarcoma literature, aiming to illuminate the treatment path for this condition^13^.

## MATERIALS AND METHODS

A systematic review was done through a comprehensive search of pertinent data in the following databases: MEDLINE, Cochrane, EMBASE, and Google Scholar. Additionally, reference lists of relevant studies were meticulously examined. The search terms encompassed keywords such as amputation, osteosarcoma, bone cancer, recurrence, and metastasis, with the inquiry containing research up until 2021.

Inclusion criteria involved studies conducting a comparative analysis between LSS and amputation groups, focusing on osteosarcoma patients (primary, central, and high grade) across all four limbs and possessing substantial datasets about local recurrence or five-year overall survival rates. On the contrary, studies falling below the threshold of case series with less than 20 patients, along with letters, case reports, editorials, or reviews lacking comparative data on LSS or amputation groups, were excluded. Pathological fractures were not evaluated, as this was not the objective of our study and none of the included studies reported their occurrence in their populations. Objective measures established by surgeons were employed to gather clinical outcomes during the latest follow-up.

The results extracted from the gathered articles were methodically organised. The authors J. R. and I. G. E. W devised a structured table, meticulously integrating all relevant information into a dedicated database. Each journal was then appraised, and a consensus was reached among the authors if these met the standard quality. Vital details, including the lead author's name, publication year, study design, patient count within each group, patients' demographic characteristics such as age and gender, local recurrence frequencies, and five-year overall survival rates, were extracted from the articles by the predefined inclusion criteria.

## RESULTS

**Fig. 1:**
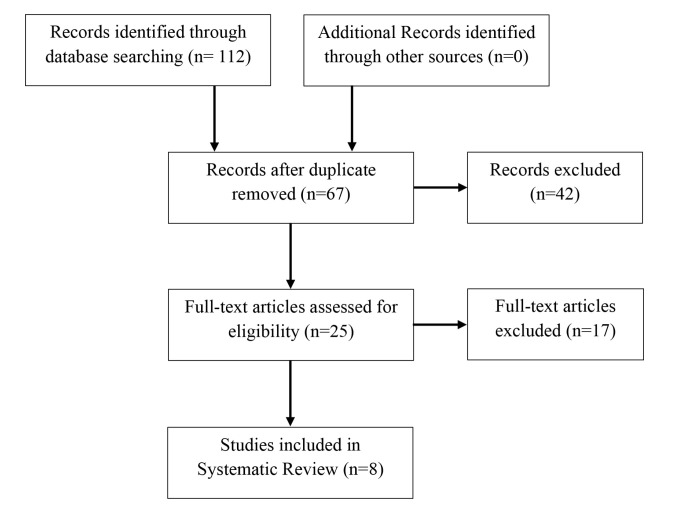
Flow chart of studies included and excluded.

A total of eight articles from peer-reviewed journal were preliminarily identified as potentially fitting the criteria and subsequently subjected to thorough scrutiny through full-text examination. Out of the initial pool, 112 articles were excluded based on adherence to the predefined inclusion criteria. In comparison, an additional 59 articles were disregarded due to their overlap in terms of the patient population studied. This assessment was grounded in institution affiliation, patient count, and authorship. Ultimately, eight articles remained^15-21^, aligning squarely with the inclusion parameters, thus warranting their incorporation into the analysis. A detailed visual representation of this meticulous screening and selection process can be found in Fig. 1.

All the studies deemed eligible for consideration adopted a retrospective approach and were predominantly confined to a single institution. The studies that were included were held from years 1983 to 2015 but published from 1990 to 2019. The collective sample contained 1389 patients, with study sizes ranging from 22 to 355 patients. The spectrum of osteosarcoma according to Enneking staging extended from IIA to IIB, with one study not furnishing stage-specific data. Notably, the studies did not explicitly outline disparities in patient staging between the two groups under investigation. The core attributes of the studies engaged are concisely summarised in Table I^8,15-21^. Table II^8,15-21^ shows the outcome of the studies, whereas Table III^8,15-21^. encapsulates the comprehensive details of the studies incorporated within the systematic review.

**Table I:** General characteristics of included studies in the analysis.

**No**	**References**	**Study Period Number**	**Patient (Male /Female)**	**Sex (Range)**	**Median Age Stage**	**Enneking**	**Country**
1	Xu *et al,* 2018^8^	2000-2015	89	50/39	Age Group:	n/a	China
				<20: 30 patients			
				20-40: 39 patients			
				>40: 20 Patients			
2	Bacci *et al,* 1990^15^	1983-1986	127	72/55	15 (5-43)	Stage IIB	Italy
3	Gherlinzoni *et al,* 1992^16^	1983-1988	355	239/116	n/a	Stage IIA	Italy
4	Bacci *et al*, 1993^17^	1986-1989	164	89/75	n/a	Stage IIB	Italy
5	Bacci *et al,* 2001^18^	1983-1995	234	137/97	n/a	Stage IIB	Italy
6	Fujiwara *et al,* 2019^19^	2007-2015	226	136/90	15 (4-67)	Stage IIB	United Kingdom
7	Hegyi *et al,* 2011^20^	1988-2006	172	65/57	13.8 (5.5-17.6)	Stage IIB	Hungary
8	Liao *et al,* 2018^21^	1990-2016	22	15/7	55.5 (21-72)	Stage IIA	China

**Table II:** Characteristic of outcome of studies.

**No**	**References**	**Median Disease- Free Survival Surgery / Amputation)**	**Local Recurrence (Limb Sparing Amputation)**	**5-year Survival (Limb Sparing Surgery / Surgery / Amputation)**	**Metastatic Occurrence Occurrence (Limb Sparing**	**Follow-up (Range)**
1	Xu *et al,* 2018^8^	n/a	17/10	n/a	n/a	n/a
2	Bacci *et al,* 1990^15^	14 (2-96)	5.5/3.1	69/51	53/51	47 (30-69)
3	Gherlinzoni *et al,* 1992^16^	20 (5-88)	19/13	67/56	87/67	51 (19-88)
4	Bacci *et al,* 1993^17^	22.9 (10-49)	2/1	71/63	6/4	54 (36-76)
5	Bacci *et al,* 20011^8^	n/a	30/1	62/48	161/46	23.8 (2-96)
6	Fujiwara *et al,* 2019^19^	n/a	n/a	n/a	n/a	61 (6-120)
7	Hegyi *et al,* 2011^20^	n/a	n/a	82/30	n/a	n/a
8	Liao *et al,* 2018^21^	n/a	n/a	n/a	n/a	n/a

**Table III:** Studies included in the analysis.

**No**	**References**	**Journal**	**Study Design**	**Level of Evidence**
1	Xu *et al,* 2018^8^	Clinical Orthopaedics and	Cohort	III
		Related Research		
2	Bacci *et al,* 1990^15^	Cancer	Randomised Controlled Trial	I
3	Gherlinzoni *et al,* 1992^16^	Annals of Oncology	Randomised Controlled Trial	I
4	Bacci *et al,* 1993^17^	Cancer	Randomised Controlled Trial	I
5	Bacci *et al,* 2001^18^	European Journal of Cancer	Randomised Controlled Trial	I
6	Fujiwara *et al,* 2019^19^	The Bone and Joint Journal	Randomised Controlled Trial	I
7	Hegyi *et al,* 2011^20^	Wiley-Liss	Randomised Controlled Trial	II
8	Liao *et al,* 2018^21^	Scientific Reports	Randomised Controlled Trial	II

Within the confines of the investigation, three distinct studies encompassing 646 patients focused on appraising the 5-year DFS of individuals grappling with limb osteosarcoma. The median DFS ranged from 6 to 78 months, averaging approximately 56.9 months. Simultaneously, five studies spanning 969 patients furnished insights into the occurrence of local relapses of the disease, signifying a notably elevated incidence within the LSS category as opposed to the amputation category (73.5% versus 28.1%).

Of particular note, the analysis encompassed 6 studies scrutinising the 5-year overall survival rate across a cohort of 1141 patients. The cumulative findings suggest a more considerable prevalence of patients favouring LSS over amputation, with figures reaching 351 versus 248, respectively. On a different note, outcomes from four studies spotlighted a higher frequency of metastatic occurrences among individuals who underwent LSS than those who underwent Amputation (307 versus 168 cases).

## DISCUSSION

Over the past five decades, there has been a notable enhancement in the survival rates of osteosarcoma patients. This improvement is attributed to successful neoadjuvant chemotherapy implementation and advancements in surgical methods, substantially augmenting the overall survival rates among those with primary osteosarcoma^11,14,22^. Despite these strides, contradictory outcomes from various studies have left the impact of surgical treatment methods on survival estimates largely inconclusive. Our analysis focusing on survival and local recurrence within the limb neoadjuvant therapy context revealed a considerably higher five-year survival rate with LSS compared to amputation. Notably, there was no discernible divergence in the five-year DFS between the two groups. Despite the elevated local recurrence rate post-LSS, this disparity did not register as statistically significant within the purview of the reviewed studies. Regrettably, existing literature has not provided a clear demarcation of stage and prognostic distinctions between the two groups, elements that could sway the group comparison.

Across the majority of studies integrated into our systematic review, patients subjected to LSS exhibited a significantly elevated five-year overall survival in comparison to those opting for amputation as a treatment for limb osteosarcoma.

According to Bacci *et al,* limb-salvage procedures tend to be associated with reduced surgical margins, which can elevate the likelihood of local recurrence. Their assertion aligns with the notion that limb-salvage approaches might yield suboptimal surgical margins, subsequently contributing to heightened local recurrence rates and inferior survival outcomes. Notably, Bacci *et al's* study demonstrates that patients undergoing LSS achieved a significantly higher 5-year DFS than those undergoing amputation (63% versus 49%)^22,23^.

This trend is similarly mirrored in the findings of other studies, such as Mavrogenis *et al,* Harrison *et al,* and Sluga *et al,* which revealed that LSS-treated patients exhibited superior 5-year DFS compared to their amputation counterparts (84% versus 74%, 68-85% versus 6-70%, and 73% versus 64% and, respectively)^24-26^. Our observations were potentially influenced by the interplay of neoadjuvant chemotherapy and patient staging. Alas, the divergence in patient stages between the two groups could have influenced the outcomes, thereby introducing an element of variability. It's worth noting that advances in medical and diagnostic resources have likely contributed to improving disease-free survival rates for osteosarcoma patients. However, within our analysis, no substantial variance in DFS was detected between patients undergoing LSS or amputation procedures^26,27^.

This study was limited because we could not analyse in each stage of osteosarcoma. Most studies included all stages of osteosarcomas. This study included seven randomised control trials and one cohort studies. Although, we have tried to minimalise the bias using more RCTs, we could not use tools to assess the quality because of the study type heterogeneity.

## CONCLUSION

To sum up, this meticulous review adds valuable insights to the current literature concerning the survival and local recurrence outcomes for individuals afflicted by limb osteosarcoma, subjected to treatment via either LSS or amputation. Notably, LSS showcased a superior five-year survival rate when pitted against the amputation procedure. Despite the elevated frequency of local recurrence observed among LSS-treated patients, this phenomenon did not wield a discernible impact on the overall survival outcome. Notably, our investigation, offering the latest and most comprehensive insights into the matter, underscores the necessity for future comparative studies involving patients manifesting osteosarcomas of congruent skeletal sites and stages. This, in turn, would serve to corroborate and solidify the discerned conclusions.

## CONFLICT OF INTEREST

The authors declare no potential conflict of interest.
